# Impact of Chamber/Annealing Temperature on the Endurance Characteristic of Zr:HfO_2_ Ferroelectric Capacitor

**DOI:** 10.3390/s22114087

**Published:** 2022-05-27

**Authors:** Yejoo Choi, Changwoo Han, Jaemin Shin, Seungjun Moon, Jinhong Min, Hyeonjung Park, Deokjoon Eom, Jehoon Lee, Changhwan Shin

**Affiliations:** 1 Department of Electrical and Computer Engineering, Sungkyunkwan University, Suwon 16419, Korea; mirmail9206@skku.edu (Y.C.); cwhan0105@g.skku.edu (C.H.); scott93@skku.edu (S.M.); hyun7256@g.skku.edu (H.P.); 2 Department of Electrical Engineering, University of Notre Dame, Notre Dame, IN 46556, USA; jshin6@nd.edu; 3 Department of Materials Science and Engineering, University of Michigan, Ann Arbor, MI 48109, USA; jinhongm@umich.edu; 4 School of Advanced Materials Sciences and Engineering, Sungkyunkwan University, Suwon 16419, Korea; vish0316@skku.edu (D.E.); iryes4832@skku.edu (J.L.); 5 School of Electrical Engineering, Korea University, Seoul 02841, Korea

**Keywords:** ferroelectric capacitor, hafnium zirconium oxide, chamber temperature, polarization, endurance

## Abstract

The endurance characteristic of Zr-doped HfO_2_ (HZO)-based metal–ferroelectric–metal (MFM) capacitors fabricated under various deposition/annealing temperatures in the atomic layer deposition (ALD) process was investigated. The chamber temperature in the ALD process was set to 120 °C, 200 °C, or 250 °C, and the annealing temperature was set to 400 °C, 500 °C, 600 °C, or 700 °C. For the given annealing temperature of 700 °C, the remnant polarization (2P_r_) was 17.21 µC/cm^2^, 26.37 µC/cm^2^, and 31.8 µC/cm^2^ at the chamber temperatures of 120 °C, 200 °C, and 250 °C, respectively. For the given/identical annealing temperature, the largest remnant polarization (P_r_) was achieved when using the chamber temperature of 250 °C. At a higher annealing temperature, the grain size in the HZO layer becomes smaller, and thereby, it enables to boost up P_r_. It was observed that the endurance characteristics for the capacitors fabricated under various annealing/chamber temperatures were quite different. The different endurance characteristics are due to the oxygen and oxygen vacancies in ferroelectric films, which affects the wakeup/fatigue behaviors. However, in common, all the capacitors showed no breakdown for an externally applied pulse (up to 10^8^ cycles of the pulse).

## 1. Introduction

Ferroelectric materials have been widely used/adopted for various types of sensors and devices. Among various ferroelectric materials, HfO_2_-based ferroelectric devices have attracted great interest [1]. A HfO_2_-based ferroelectric film with fluorite structure solved the drawbacks in the conventional perovskite–structure ferroelectrics. They have extraordinary compatibility with complementary metal-oxide semiconductors (CMOS) and excellent ferroelectricity at ultra-thin (<10 nm) thickness [2,3]. The ferroelectric properties of HfO_2_-based film originated from the non-centrosymmetric orthorhombic phase (o-phase), and the stabilization of the o-phase enhances the ferroelectric behavior [4]. The ferroelectric phase can be stabilized through annealing, and it can be characterized differently by various factors, such as dopant, thickness of ferroelectric film, and deposition temperature [5,6].

Various dopants with HfO_2_ have been studied, and among them, zirconium (Zr) was chosen as the most promising material for memory and logic devices [6,7]. Unlike other dopants remaining stable at much lower concentration, Zr dopants can be stable with the same percentage as Hf in HZO film. Moreover, ferroelectric properties using Zr dopants can be obtained in much lower annealing temperature (T_A_) than other dopants.

As a memory device, the ferroelectric films require good endurance properties. The main factor affecting the endurance is the oxygen vacancy (V_O_) in ferroelectric films. During the electric field cycling, V_O_ is redistributed, which results in the uniform distribution of V_O_ in the bulk region of the ferroelectric layer [8]. This phenomenon increases the P_r_ value by decreasing the built-in field and then decreases the P_r_ value with additional cycling. This increase in P_r_ is called the “wake-up effect” and the decrease in P_r_ is called the “fatigue effect” [9,10]. The breakdown of the films is observed when V_O_ forms a filament, which results as a leakage path. Therefore, it is very important to control the amount of V_O_ for the reliability of memory devices.

The characteristics of ferroelectric films can vary through various methods such as adjusting the doping effect and the chamber temperature during ALD [11,12]. Adjusting the temperature of the chamber during ALD changes the deposition rate and the average grain size of the film [6,13]. These factors change the distribution of V_O_, which can significantly affect the ferroelectricity and the endurance properties. However, studies on the relationship of the effects of chamber temperature and the endurance performance are still lacking.

In this study, the ferroelectric properties of TiN/HZO/TiN capacitors with different chamber temperatures were investigated. The electrical characteristics of each capacitor were analyzed through polarization–voltage (P-V) curves and leakage current–voltage (I-V) curves. Moreover, the endurance performance related to the amount of V_O_ was investigated under different chamber temperatures.

## 2. Fabrication

**Figure 1:**
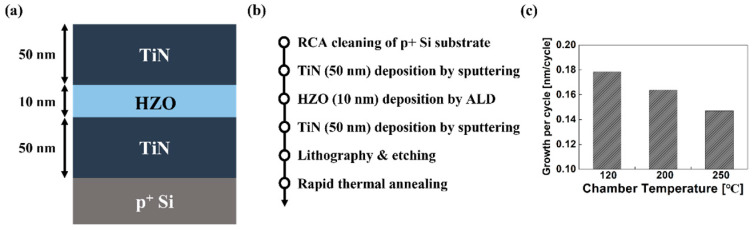
(**a**) Illustrated cross-sectional view of the fabricated TiN/HZO/TiN capacitor, and (**b**) its fabrication process flow. The HZO layer were deposited under three different temperatures in chamber, i.e., 120 °C, 200 °C, and 250 °C. (**c**) Growth per cycle of HZO film for three different chamber temperatures. Note that the growth per cycle is decreased with increasing the chamber temperature, which is due primarily to the decreasing contribution of surface exchange reaction [13].

The illustrated cross-sectional view and fabrication flow of TiN/HZO/TiN capacitors are shown in Figure 1a,b, respectively. First, a p^+^-doped silicon wafer was cleaned by a SPM cleaning, which was followed by the conventional RCA method (i.e., SC-1 cleaning and SC-2 cleaning). Then, the 50 nm-thick TiN bottom electrode was deposited on the Si substrate by using DC sputtering. The 10 nm-thick HZO thin film was deposited by thermal atomic layer deposition (ALD). The tetrakis (ethylmethylamino) hafnium (TEMAH), tetrakis (ethylmethylamino) zirconium (TEMAZ), and H_2_O source precursor were used for the ALD process to deposit the HZO film. The Hf and Zr was deposited using an ALD supercycle [14]. The aforementioned fabrication was identically completed but at three different chamber temperatures (T_CH_) of 120 °C, 200 °C, and 250 °C. As shown in Figure 1c, the growth per (super)cycle of HZO film decreased as the chamber temperature (T_CH_) increased. The total number of supercycles to make 10 nm-thick HZO thin film was set to 56, 61, and 68 for T_CH_ of 120 °C, 200 °C, and 250 °C, respectively. The 50 nm-thick TiN top electrode on the HZO layer was deposited using the DC sputtering used for bottom electrodes. Then, the HZO capacitors were patterned to have the electrode area of 6400 µm^2^. Finally, the post-metallization annealing (PMA) was completed by rapid thermal annealing (RTA) at 400, 500, 600, and 700 °C for 30 s in N_2_ atmosphere to crystallize the HZO films.

To investigate the electrical characteristics of HZO capacitors, P-V curves and I-V curves were measured using semiconductor parameter analyzer (Keithley 4200-SCS). The X-ray photoelectron spectroscopy (XPS), transmission electron microscopy (TEM), and energy-dispersive X-ray spectroscopy (EDS) were used to characterize the HZO capacitor.

## 3. Results and Discussion

### 3.1. Electrical Characteristics

**Figure 2:**
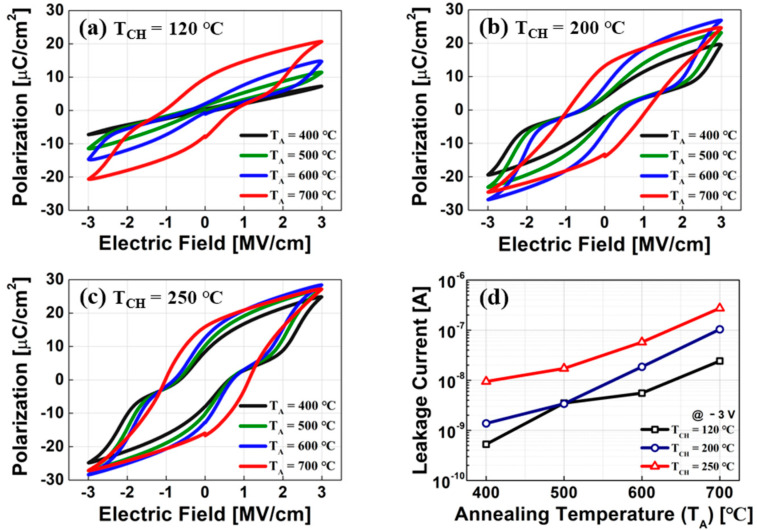
Measured polarization vs. electric field of TiN/HZO/TiN capacitor in which the HZO layer was deposited at the chamber temperature of (**a**) 120 °C, (**b**) 200 °C, and (**c**) 250 °C. Note that four different annealing temperatures (T_A_ = 400~700 °C) were used for better implementing the ferroelectric characteristics of the HZO layer. (**d**) Measured leakage current-vs.-annealing temperature for three different chamber temperatures. Note that the leakage current was measured with the voltage of −3 V across the ferroelectric capacitor.

Figure 2a–c show the measured P-V curves of the TiN/HZO/TiN capacitors with a few annealing temperatures (i.e., 400, 500, 600, and 700 °C) at T_CH_ of 120 °C, 200 °C, and 250 °C, respectively. The measured P_r_ was increased at a higher T_A_ for all the capacitors. This is mainly because the ratio of o-phase in the HZO layer was increased at the higher T_A_ [2,10]. For a given T_A_, a higher P_r_ was implemented at a higher T_CH_. This achievement was physically originated from a few reasons, as follows: (1) The number of supercycles increases at a higher T_CH_ because the deposition rate of HZO film is decreased at a higher T_CH_ (see Figure 1c). This leads to increasing the number of HfO_2_/ZrO_2_ nanolaminates, resulting in the improved P_r_ [15]. (2) The average grain size of HZO film becomes smaller at a higher deposition temperature, if H_2_O is used as the oxygen source in ALD [13]. The smaller average grain size enables us to boost up the ferroelectric polarization [10,16]. This is primarily originated from the tetragonal phase (t-phase) and the orthorhombic phase (o-phase), which are easily stabilized in smaller grain regions. (3) Because defects (noted as V_O_) are likely to be accumulated along the grain boundaries, the smaller grain size is likely to build more grain boundaries for a given volume. This would distort the distribution of V_O_. Herein, it is noteworthy that the well-balanced distribution of V_O_ in HZO films should help stabilize the ferroelectric phase, and thereby, the ferroelectric polarization would be enhanced [10,17].

In Figure 2d, the leakage current of TiN/HZO/TiN capacitors fabricated at different T_A_ and T_CH_ was measured. It turned out that for a given T_CH_ (T_A_), the leakage current increases at a higher T_A_ (T_CH_). In the MFM ferroelectric capacitor, the leakage current is mostly originated from V_O_ (i.e., the higher V_O_ is, the more the leakage current flows [15]). In Section 3.2, with TEM, XPS, and EDS data, it is investigated how V_O_ is varied at different T_CH_.

### 3.2. XPS, TEM, and EDS

**Figure 3:**
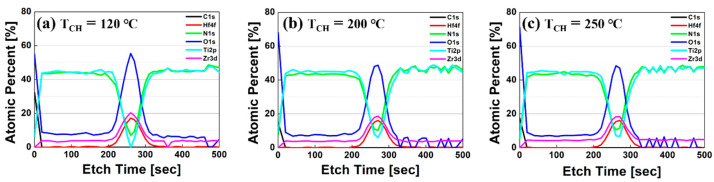
Measured XPS depth profiles of TiN (50 nm)/HZO (10 nm)/TiN (50nm) capacitor, which was fabricated at three different chamber temperatures: (**a**) 120 °C, (**b**) 200 °C, and (**c**) 250 °C.

**Figure 4:**
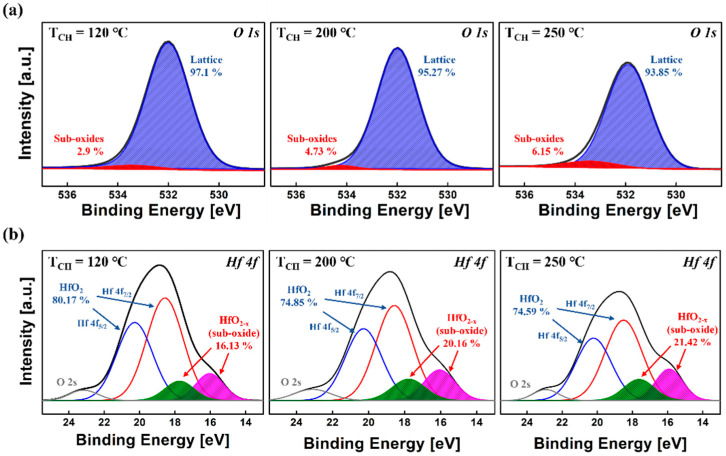
Measured (**a**) XPS O 1s spectra and (**b**) XPS Hf 4f spectra of the HZO film in TiN/HZO/TiN capacitor, which was fabricated at three different chamber temperatures (i.e., 120 °C, 200 °C and 250 °C). The O 1s levels were deconvoluted into two peaks, i.e., lattice oxygen peak and sub-oxide peak. The Hf 4f levels were deconvoluted into five peaks, i.e., HfO_2_ peaks, sub-oxide peaks, and O 2s peak. Each intensity level was normalized to the same scale.

Figure 3 shows the XPS depth profile of the TiN/HZO/TiN capacitor at T_A_ of 700 °C under three different T_CH_ values. The atomic percent clearly reveals the TiN/HZO/TiN device structure. Among many atoms in the profile, the O 1s in the HZO layer is deconvoluted to figure out the oxygen bonds in great detail (see Figure 4). Notice that the intensities of each net peak were normalized to the same scale. The O 1s was divided into two peaks (i.e., lattice oxygen peak and sub-oxide peak). The lattice oxygen peak describes the bonds such as Hf-O and Zr-O, of which the binding energy is located in ≈532 eV. The sub-oxide peak indicates oxygen in a lattice, which does not contain its full complement of oxygen (and therefore, indicating the presence of vacancies). The peak describes the bonds such as oxygen interstitial and oxygen vacancy (V_O_), of which the binding energy locates at ≈534 eV.

The amount of O 1s in Figure 3 and Figure 4a confirms that there was a difference in the ratio of O 1s in HZO film, depending on three different T_CH_. The intensity of peak amplitude is decreased at a higher T_CH_. In addition, the atomic percent of Ti and N is decreased at a lower T_CH_ (see Figure 3). In other words, the formation of the dead layer (i.e., TiO_2_ and TiON) between the HZO layer and the TiN electrode was suppressed at a lower T_CH_, which reduces the amount of V_O_ in the HZO layer. It was confirmed that the ratio of V_O_ increases if the dead layer between the HZO layer and the TiN electrode is formed [18].

It was observed that the percentage of sub-oxide bonding is increased at a higher T_CH_ (see Figure 4a). This also indicates that the ratio of V_O_ increases. In other words, the oxygen should move from the HZO layer to the electrodes to make the interfacial layer, and thereby, the V_O_ in HZO film is increased [17]. Hence, the increased V_O_ means that the larger amount of oxygen has moved toward the interfacial layer. In addition, in the XPS for Hf 4f spectra, it was confirmed that the sub-oxide means the oxygen vacancy in the HZO layer [19]. As shown in Figure 4b, it was observed that the ratio of sub-oxide in Hf 4f spectra is increased when T_CH_ is increased, which leads to a higher V_O_. In summary, the ratio of oxygen in the HZO layer becomes smaller at a higher T_CH_, which is closely associate with the amount of V_O_. A higher T_CH_ would make the ratio of V_O_ higher. This is well agreed to the increase in leakage current at a higher T_CH_ because of the increased V_O_ (see Figure 2d).

**Figure 5:**
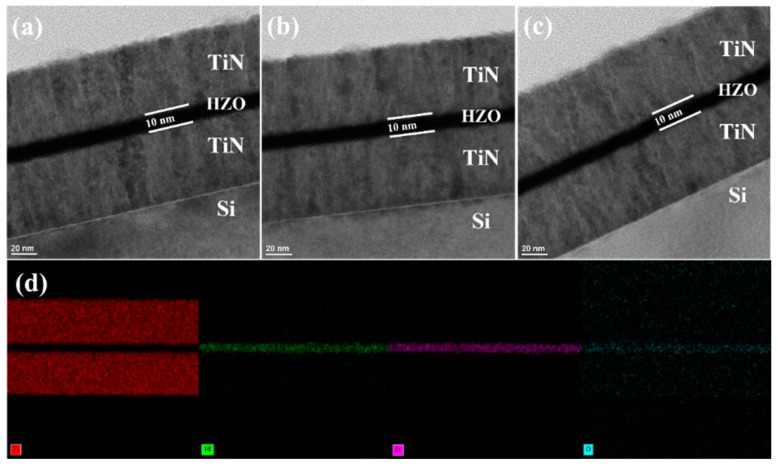
The transmission electron microscopy (TEM) image of the TiN/HZO/TiN capacitor, which was fabricated at three different chamber temperatures: (**a**) 120 °C, (**b**) 200 °C, and (**c**) 250 °C. (**d**) The energy-dispersive spectroscopy (EDS) images for titanium, hafnium, zirconium, and oxygen in the HZO capacitor at the chamber temperature of 250 °C.

Figure 5a–c show the TEM image of TiN/HZO/TiN capacitors fabricated at three different chamber temperatures (T_CH_) with T_A_ of 500 °C. It was observed that the thicknesses of the HZO films were all the same: 10 nm. Figure 5d shows the EDS image of the TiN/HZO/TiN capacitor fabricated with T_A_ of 700 °C. The ratio of elements in each layer can be determined through the EDS analysis (see Table 1). For example, the largest (smallest) atomic rate of oxygen in HZO films was implemented with T_CH_ of 120 °C (250 °C), which was consistent with the XPS analysis. The amount of oxygen vacancy (V_O_) was increased, as the oxygen was decreased with increasing T_CH_. However, the discrepancy of the ratio of elements exists between the XPS and EDS analysis. Note that the purpose of the EDS analysis in this work was to confirm/verify the change of oxygen atoms. 

### 3.3. Endurance

**Figure 6:**
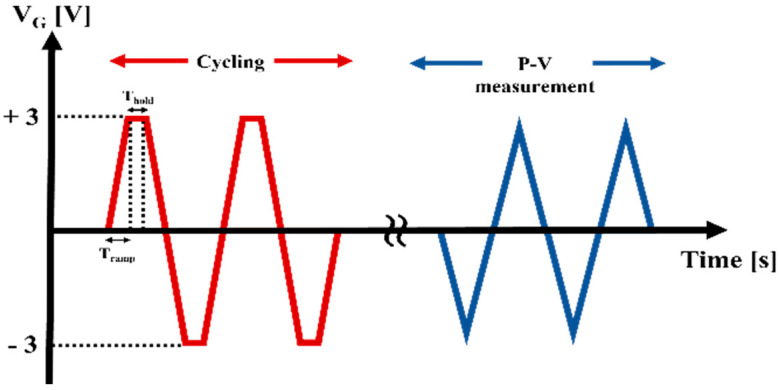
Illustrated pulsing scheme for analyzing the wake−up and fatigue behaviors of TiN/HZO/TiN capacitor. The cycling was first completed using the trapezoidal pulse with the ramp time (T_ramp_) of 1 µs and the hold time (T_hold_) of 1 µs. Afterwards, the P-V measurement was made using the triangular pulse. Note that the amplitude for both pulses is set to 3 V.

**Figure 7:**
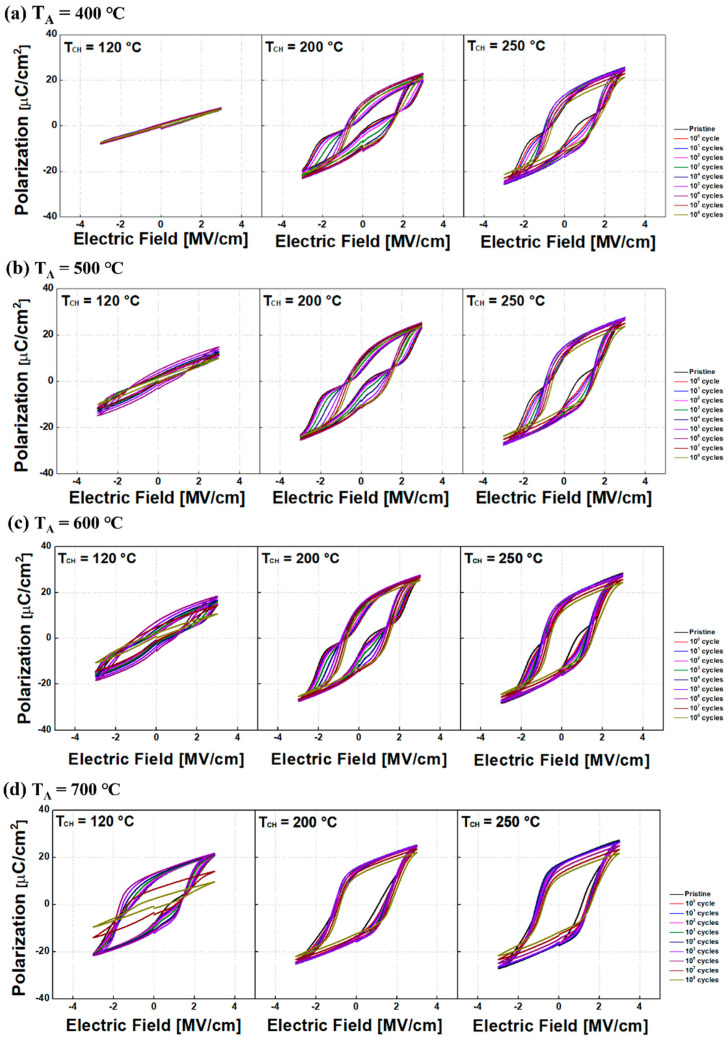
Measured polarization vs. electric field (PE) characteristics of TiN/HZO/TiN capacitors fabricated at various chamber temperatures (i.e., 120 °C, 200 °C, and 250 °C) as well as at various annealing temperatures (T_A_): (**a**) 400 °C, (**b**) 500 °C, (**c**) 600 °C, and (**d**) 700 °C. Note that the different numbers of cycles were used to explore the endurance characteristics of the ferroelectric capacitor.

The endurance (especially, affected by an electric field cycling) in TiN/HZO/TiN capacitors was investigated. Figure 6 shows the pulsing scheme for evaluating the endurance of the capacitors. The electric field cycling was completed with using a trapezoidal pulse, and the number of cycling was applied up to 10^8^. After the cycling pulses were applied, a triangular pulse was applied to measure the P-V characteristic of the capacitor. Note that both pulses have the same peak amplitude of 3 V. The P-V characteristic of each capacitor was compared to each other (see Figure 7).

The remnant polarization (P_r_) of each HZO capacitor in pristine state is as follows: In case of T_CH_ of 120 °C, P_r_ of 0.28 µC/cm^2^, 1.17 µC/cm^2^, 2.68 µC/cm^2^, and 17.21 µC/cm^2^ was observed for T_A_ of 400 °C, 500 °C, 600 °C, and 700 °C, respectively. In case of T_CH_ of 200 °C, P_r_ of 5.66 µC/cm^2^, 6.88 µC/cm^2^, 13.43 µC/cm^2^, and 26.37 µC/cm^2^ was observed for T_A_ of 400 °C, 500 °C, 600 °C, and 700 °C, respectively. Finally, in case of T_CH_ of 250 °C, P_r_ of 16.38 µC/cm^2^, 20.46 µC/cm^2^, 25.3 µC/cm^2^, and 31.8 µC/cm^2^ was observed for T_A_ of 400 °C, 500 °C, 600 °C, and 700 °C, respectively. Regardless of T_CH_, it turned out that P_r_ was increased with increasing T_A_. For a given/identical T_A_, P_r_ can be improved with higher T_CH_. This is primarily because the amount of ferroelectric phase was increased with increasing either T_A_ or T_CH_ [2,9].

**Figure 8:**
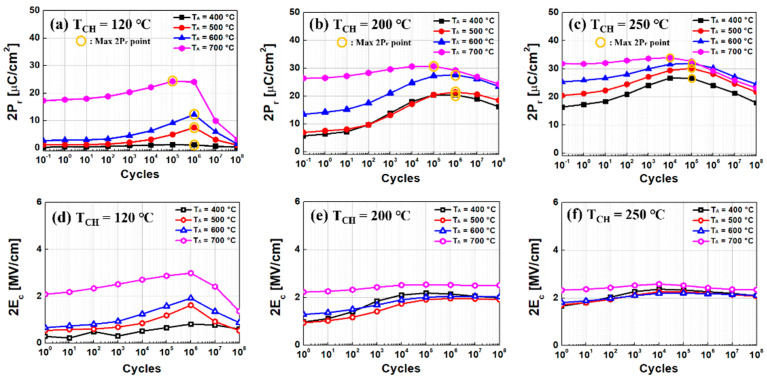
Measured representative ferroelectric characteristics (i.e., remnant polarization (2P_r_) and/or coercive electric field (2E_c_)) versus cycles, for given three different chamber temperatures, (**a**,**d**) 120 °C, (**b**,**e**) 200 °C, and (**c**,**f**) 250 °C with four different annealing temperatures (T_A_ from 400 °C to 700 °C). The yellow−colored circle indicates the max point of 2P_r_.

As shown in Figure 8, all the capacitors did not show any breakdown up to 10^8^ cycles. However, depending on T_CH_, the number of cycles at which the fatigue begins (i.e., P_r_ is about to decrease) is varied. Regardless of T_CH_, higher P_r_ was observed with higher T_A_. This is primarily originated from a well-distributed V_O_ in bulk region of HZO film at a higher T_A_ [10]. 

**Figure 9:**
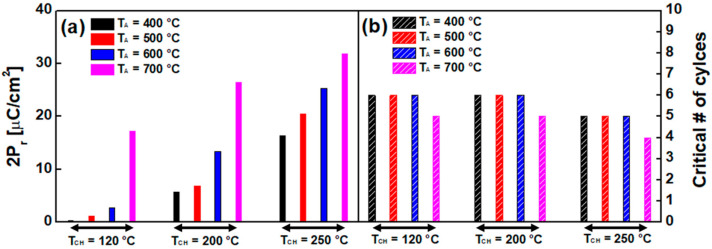
(**a**) Maximum remnant polarization (2P_r_) and (**b**) critical number of cycles at which fatigue begins for given three different chamber temperatures, 120 °C, 200 °C, and 250 °C, with four different annealing temperatures (T_A_ from 400 °C to 700 °C).

It is known that the ferroelectric characteristics (notably, represented by P_r_) can be improved as the number of cycles increases (a.k.a., wake-up effect). However, the ferroelectric characteristics should not be ever enhanced because of fatigue. The maximum value of 2P_r_ and the number of cycles at which fatigue begins (a.k.a. critical number of cycles) are summarized in Figure 9. It turned out that the value of 2P_r_ increases with increasing T_A_. (However, only for T_CH_ of 120 °C, T_A_ of 400 °C shows insufficient ferroelectricity, which results in a low 2P_r_.) Compared to the other T_CH_ of 200 °C and 250 °C, the measured P_r_ was significantly degraded for T_CH_ of 120 °C. When H_2_O reactant is used as an oxygen source in ALD, the lowest T_CH_ (i.e., 120 °C) forms the largest average grain size. This causes more formation of monoclinic phase (m-phase), which is non-ferroelectric phase, and it makes the depolarization field stronger [20,21]. Therefore, for T_CH_ of 120 °C, the sudden decrease in P_r_ in the HZO capacitor was understood with less m-phase.

In Figure 9, it was also observed that the critical number of cycles (i.e., the number of cycles at which fatigue begins) decreases at a higher T_CH_ for the same T_A_. As shown in Figure 2d and Figure 4, more V_O_ in the ferroelectric layer was observed at a higher T_CH_, and thereby, so is the leakage current in HZO capacitor (this is because the excessive amount of V_O_ makes undesirable conducting paths in the HZO film [10,22]). The more V_O_ in the ferroelectric film should cause the ferroelectricity of the film to be fatigued at a rapid pace.

## 4. Conclusions

In this work, the impact of chamber/annealing temperatures in the atomic layer deposition (ALD) process on the ferroelectric property of the HZO layer in a TiN/HZO/TiN capacitor was investigated. Regardless of the chamber temperature (T_CH_), a higher remnant polarization (P_r_) was achieved with a higher annealing temperature (T_A_). For a given T_A_, P_r_ was increased with increasing T_CH_. This ferroelectricity was well retained up to the cycles of 10^8^. However, the capacitors fabricated under the three different T_CH_ showed different endurance performances in terms of the critical number of cycles. This is due primarily to the oxygen and oxygen vacancies in the HZO layer (which was quantitatively analyzed and confirmed by XPS and EDS analysis). The more oxygen vacancies (V_O_) at a higher T_CH_ enabled for P_r_ to be improved, but they made an undesirable conducting path in the HZO film, resulting in decreasing the critical number of cycles.

## Figures and Tables

**Table 1:** Energy-dispersive spectrometer (EDS) quantification of elements in HZO layer.

Chamber Temperature (T_CH_) (°C)	Atomic Percent (%)					
	Ti	N	Hf	Zr	O	Si
120	4.50	9.76	20.90	18.16	30.44	16.24
200	9.20	4.49	24.95	21.00	23.32	17.03
250	10.74	10.80	24.75	21.18	19.38	13.15

## Data Availability

The data presented in this study are available on request from the corresponding author.
